# Measuring cognitive load

**DOI:** 10.1007/s40037-017-0395-4

**Published:** 2018-01-05

**Authors:** John Sweller

**Affiliations:** School of Education, University of New South Wales, Sydney, Australia

The paper ‘Cognitive load predicts point-of-care ultrasound simulator performance’ by Aldekhyl, Cavalcanti, and Naismith, in this issue of Perspectives on Medical Education [1], is an important paper that adds to work on cognitive load theory and medical education [2–4]. The implications of the findings of this paper extend substantially beyond the confines of medical practice that is the focus of the work. In this commentary, I will discuss issues associated with obtaining measures of cognitive load independently of content task performance during instruction. I will begin with a brief history of attempts to provide independent measures of cognitive load.

In the 1980s, cognitive load was used as a theoretical construct to explain experimental results with very little attempt to directly measure load [5]. The theory was used to predict differential learning using particular instructional designs. Randomized controlled trials were run to test the predictions and if the hypothesized results were obtained they were attributed to cognitive load factors. The distinction between extraneous and intrinsic cognitive load had not been specified but the results were due to what was called and continues to be called extraneous cognitive load. Cognitive load was an assumed rather than a measured construct. At that time, the only attempt to provide an independent indicator of load was to use computational models [6] with quantitative differences between models used as cognitive load proxies.

The first rating scale measure of cognitive load was introduced in the early 1990s by Fred Paas [7]. The Paas scale continues to be the most popular measure of cognitive load and was used by Aldekhyl et al. to validate alternative measures of load. It is very easy to use and requires no more than a minute or so of a participant’s time. Used primarily to measure extraneous cognitive load it has repeatedly indicated that instructional designs hypothesized to decrease cognitive load as measured by the scale, increase performance test scores [8].

The Paas scale does not distinguish between categories of cognitive load and so when intrinsic load was introduced, the scale could just as easily be used to successfully measure intrinsic load. Intrinsic cognitive load was introduced because it was noticeable that cognitive load effects attributed to extraneous cognitive load were not obtainable using simple, low element interactivity information. Cognitive load theory relies on the information being processed imposing a high cognitive load. If it is not high, the results predicted by the theory are unlikely to be obtained.

There are alternatives to the Paas scale. Secondary tasks that require learners to engage in another task that is secondary to the primary task is the most common alternative [9, 10]. Reduced performance on a secondary task indicates an increased working memory load imposed by the primary task. In addition, attempts have been made to develop psychometric scales that distinguish between categories of cognitive load [11]. Such scales rely on learners being able to distinguish between extraneous and intrinsic cognitive load, which may not always be possible. If, for example, learners are unaware that physically integrated instruction is easier to process than split-source instruction, any difficulty they face is likely to be attributed to intrinsic rather than extraneous cognitive load, leading to a failure of the scales to distinguish between the two sources of cognitive load.

The Aldekhyl et al. paper does not attempt to distinguish between extraneous and intrinsic cognitive load but instead is concerned solely with intrinsic load that is varied by altering levels of expertise. Of interest, the procedures described could just as easily be used to distinguish between levels of extraneous load.

As indicated above, the paper is important not only because it describes new measures of cognitive load relevant to medical education and medical practice, but because the measures seem likely to be applicable in a variety of areas. The major finding is the negative relation between gaze shift rate and the Paas scale. It may be reasonable to speculate that decreases in intrinsic cognitive load due to increases in expertise allow people to spend less time on particular aspects of an image and rapidly shift their gaze to other important aspects of the image. A rapid shifting of gaze may indicate a rapid assimilation of essential information. The importance cannot be overestimated of a cognitive load measure that reflects the speed that information is assimilated.

While this finding has relevance when medical practitioners scan an ultrasound image, it is likely to be equally important when people scan any image or perform in a real task environment while learning. If so, it is a finding that may have considerable generality and importance that deserves follow-up studies.
